# Curved singular beams for three-dimensional particle manipulation

**DOI:** 10.1038/srep12086

**Published:** 2015-07-13

**Authors:** Juanying Zhao, Ioannis D. Chremmos, Daohong Song, Demetrios N. Christodoulides, Nikolaos K. Efremidis, Zhigang Chen

**Affiliations:** 1 The MOE Key Laboratory of Weak-Light Nonlinear Photonics, and TEDA Applied Physics Institute and School of Physics, Nankai University, Tianjin 300457, China; 2 CREOL/College of Optics, University of Central Florida, Orlando, Florida 32816; 3 Department of Physics and Astronomy, San Francisco State University, San Francisco, CA 94132; 4 Science and Technology on Solid-State Laser Laboratory, North China Institute of Electronics Optics, Beijing 100015, China; 5 Department of Mathematics and Applied Mathematics, University of Crete, Heraklion 71409, Greece; 6 Max Planck Institute for the Science of Light, Erlangen D-91058, Germany

## Abstract

For decades, singular beams carrying angular momentum have been a topic of considerable interest. Their intriguing applications are ubiquitous in a variety of fields, ranging from optical manipulation to photon entanglement, and from microscopy and coronagraphy to free-space communications, detection of rotating black holes, and even relativistic electrons and strong-field physics. In most applications, however, singular beams travel naturally along a straight line, expanding during linear propagation or breaking up in nonlinear media. Here, we design and demonstrate diffraction-resisting singular beams that travel along arbitrary trajectories in space. These curved beams not only maintain an invariant dark “hole” in the center but also preserve their angular momentum, exhibiting combined features of optical vortex, Bessel, and Airy beams. Furthermore, we observe three-dimensional spiraling of microparticles driven by such fine-shaped dynamical beams. Our findings may open up new avenues for shaped light in various applications.

Shaping the phase and amplitude of a light field in order to promote certain desired features during propagation has become nowadays increasingly interesting and demanding, across several disciplines. For instance, considerable research activity has been directed toward the fundamental physics and applications of singular beams, including optical vortices and high-order Bessel beams carrying orbital angular momentum (OAM)^1^,^2^,^3^,^4^,^5^,^6^,^7^,^8^,^9^,^10^,^11^,^12^,^13^,^14^,^15^,^16^,^17^,^18^. Such singular beams typically propagate along straight trajectories. Another example is that of self-accelerating Airy beams that can travel along curved trajectories^19^, which have recently been highly touted and tested in numerous applications such as optical micromanipulation^22^,^23^, routing of surface plasmon polaritons^24^,^25^ and electrons^26^, as well as optical high-resolution imaging^27^. Although optical vortex, Bessel, and Airy beams have each played a unique role in the arena of trapping and manipulation, combining their features (OAM, diffraction-free, and self-acceleration) into one light entity has been long-sought-after, and yet, to our knowledge, never been achieved. Despite all the efforts devoted to shaping a Gaussian-like beam into arbitrary trajectories^28^,^29^,^30^, it still remains a challenge to impose the OAM even onto the main lobe of an asymmetric two-dimensional Airy beam^31^. The natural question arises: is it possible to synthesize a donut-like diffraction-free self-accelerating singular beam capable of traveling along any predesigned trajectory?

In this work, we demonstrate with rigorous theoretical analysis that a judiciously shaped singular beam can be navigated along an arbitrary trajectory with a preserving OAM and a nonexpanding dark “hole” in the main lobe. We observe particular cases in which the beam can curve along parabolic, hyperbolic and even three-dimensional (3D) spiraling trajectories while its field profile asymptotically takes the form of a diffraction-resisting higher-order Bessel function. In our experiments, this class of curved singular beams, is employed to optically trap and rotate microparticles in 3D spiral trajectories under the combined action of radiation pressure, gradient force, and OAM. Because the dark “hole” maintains its shape during propagation, this type of singular beams could in principle be applied also for photophoretic manipulation^32^,^33^ of aerosols, light-absorbing particles, as well as low-index transparent microparticles.

To construct such self-accelerating singular beams that can travel along arbitrary trajectories, a new theoretical approach (see the supplementary material) is developed, going beyond that used in synthesizing a fundamental curving Bessel beam^29^,^34^. Central to this problem is to identify an appropriate phase function *Q*(*ξ*, *η*) for the input wavefront 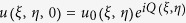. In the paraxial regime this wavefront will evolve according to the Fresnel diffraction integral:


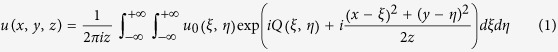


where the transverse coordinates, defined as (*x*, *y*) at the observation plane and (*ξ*, *η*) at the input plane, and the propagation distance *z* are all normalized from the real coordinates. The phase function *Q*(*ξ*, *η*), designed to produce an accelerating singular beam with a given transverse width and a topological charge (of order *m*), is the key in directing all rays in order to create a curved vortex structure along an arbitrary focal trajectory (*f*(*z*), *g*(*z*), *z*), as illustrated in Fig. 1a. Any point on this curve represents the center of the singular beam, i.e., the center of the vortex singularity embedded in the main lobe, as constructed from a bundle of skewed conical rays emanating from the same circle on the input plane. The interference of these rays leads to a high-order Bessel-like pattern with a helical phase that propagates along the predesigned path (Fig. 1b). A detailed procedure and algorithm for computing the phase function *Q*(*ξ*, *η*) is presented in the Supplementary Information S2. As we shall demonstrate below, such carefully designed beams exhibit resistance to diffraction while keeping the central main lobe and topological charge remarkably invariant.

As a typical example, we first demonstrate a singly-charged (*m* = 1) optical beam self-accelerating along a parabolic trajectory designed with the aforementioned approach. The calculated phase information is used to create a computer generated hologram^33^,^34^, as shown in Fig. 2a, and the corresponding numerical simulation of the side-view beam propagation is shown in Fig. 2b. Clearly, the beam possesses a donut-shaped transverse pattern in the main lobe surrounded by a series of rings, as seen from snapshots taken at different propagation distances (Fig. 2c–f). These results illustrate that the Bessel-like singular beam maintains a constant width in the main lobe while bending during propagation, although the overall donut pattern is slightly deformed (mainly due to the interference with rays generating the focus at smaller z) as the peak intensity circulates along the main ring. The interferogram (inset of Fig. 2f) indicates that the vortex structure of the singular beam is preserved even after more than one meter of propagation along the parabolic trajectory. To experimentally demonstrate such a self-accelerating singular beam, an expanded Gaussian beam (*λ* = 488 nm) passes through a spatial light modulator (SLM) to read out the hologram encoded with the desired phase structure at input. After reconstruction through a typical 4*f* system with spatial filtering, the beam is recorded by a CCD camera, mapping out the self-bending trajectory by the transverse snapshots taken at different distances as presented in the bottom panels of Fig. 2. Remarkably, such a bending singular beam travels along the predesigned parabolic path up to 140 cm with a fairly invariant (diffraction-resisting) main lobe and a preserving vortex (OAM) structure. These experimental observations are in good agreement with our theoretical predictions.

Using this same approach, the singular beam can be made to follow any arbitrary trajectory. Other exemplary examples include two-dimensional snake-like or hyperbolic trajectories as well as arbitrarily designed curved trajectories in 3D space. In Fig. 3, we show a triply-charged (*m* = 3) singular beam propagating along a predesigned 3D trajectory, where the white focal curve (*f*(*z*), *g*(*z*), *z*) is given by *f*(*z*) = 5 tanh[0.12(*z* − 10)] + 5, and g(*z*) = 6sech[0.12(*z* − 10)]. Careful analysis shows that the singular beam asymptotically takes the high-order (*J*_3_) Bessel profile along the curved trajectory, while the central main lobe exhibits a diffraction-resisting dark core with a preserved topological charge. The singular beam curves in both *x* and *y* directions during propagation along the longitudinal *z* direction, as shown in Fig. 3a. The transverse patterns and interferograms taken at different propagation distances (Fig. 3b–d) show clearly that the main lobe is nearly diffraction-free, and the topological charge (*m* = 3) persists during propagation. Again, the experimental results match well with those from theory. We mention that the spatial twist and OAM has also been suggested from the theory of spontaneous knotting of nonlinear self-trapped waves, but here all spatial shaping is achieved in linear regime and can be in principle implemented in free space^35^,^36^.

One of the prime motivations of intelligent beam engineering is to employ them for various applications in optical trapping and manipulation of microscopic objects^2^,^3^. To this end, we demonstrate that such self-accelerating singular beams can be implemented in an optical tweezers’ setting to actively control microparticles. Inasmuch as driving an electron into spiral motion by applying electric and magnetic fields, a self-bending Bessel-like singular beam can be used to set a transparent polystyrene bead into spiral motion due to combined action of trapping (by gradient force), pushing (by radiation pressure), and spinning (by OAM), as illustrated in the top panel of Fig. 4a. Typical experimental results of rotating trapped 2μm polystyrene beads in aqueous solution at different longitudinal positions along a curved hyperbolic secant trajectory are displayed in Fig. 4b–e, where the images of the trapped beads were taken by illuminating the sample with a white-light beam from the opposite direction of the trapping beam as in optical tweezers with bright-field microscopy. As seen from the Media 2, the beams are gradually trapped onto the annulus of maximum beam intensity, and rotated due to the transfer of OAM from the singular beam^4^. We emphasize that the beads are actually driven into a 3D spiral motion as illustrated in Fig. 4a, but are visualized in different transverse 2D planes being pushed against thin glass (Fig. 4b–e). As we move our sample along the longitudinal direction, the particles will be trapped and guided into different transverse locations along the curved path. By changing the trajectory and the order of the Bessel-like singular beam, one can in principle actively control the transporting path and rotating radius of the trapped beads.

In summary, we have theoretically and experimentally demonstrated self-accelerating optical singular beams propagating along arbitrary trajectories and their applications in optical manipulation of microparticles. These beams not only maintain a nearly invariant dark “hole” in the central main lobe but also preserve their angular momentum while curving in 2D or 3D space, exhibiting combined features of diffraction-resisting, self-acceleration and orbital angular momentum. Our findings may open up new avenues for shaped light in various applications in biological, soft-condensed matter, and atmospheric sciences. This method can also be readily extended beyond the paraxial limit to achieve large angle trajectories^21^ and various sharp bending desirable for various applications.

## Additional Information

**How to cite this article**: Zhao, J. *et al.* Curved singular beams for three-dimensional particle manipulation. *Sci. Rep.* **5**, 12086; doi: 10.1038/srep12086 (2015).

## Supplementary Material

Supplementary Information

## Figures and Tables

**Figure 1:**
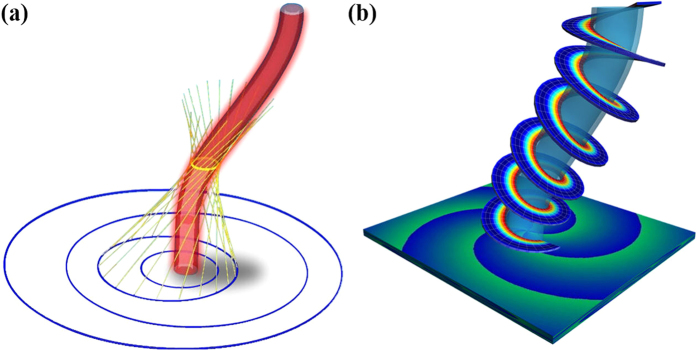
Schematic of a diffraction-resisting singular beam curving in free space. (**a**) Propagation of the beam along a curved trajectory, where rays emitted from expanding circles on the input plane are skewed but converged along a specified focal curve. (**b**) Corresponding helical phase structure of the beam along the curve. Due to the self-acceleration, the center of the singular beam shifts laterally during propagation, but its donut shape and vorticity remain intact (see Media 1).

**Figure 2:**
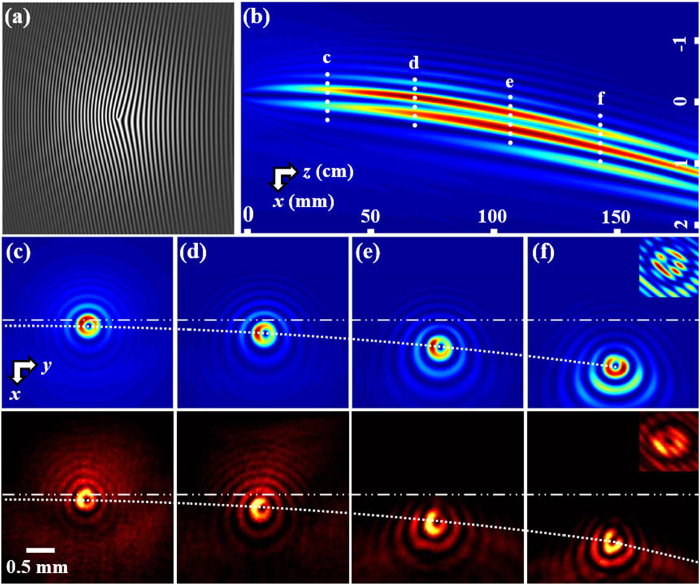
Demonstration of a singly-charged singular beam (*m* = 1) self-accelerating along a parabolic trajectory. (**a**) Computer generated hologram showing a modulated vortex phase at the input. (**b**) Numerically simulated side-view propagation of the resulting vortex beam along predesigned parabolic trajectory. (**c**–**f**) Snapshots of transverse beam patterns taken at different propagation distances marked in (**b**), where top panels are from simulation and bottom panels from experiment. The dotted white curve illustrates the bending trajectory relative to the launching direction of the initial beam. The insert in (**f**) corresponds to the interferogram of the singular beam with an inclined plane wave, showing that the vorticity is preserved along propagation.

**Figure 3:**
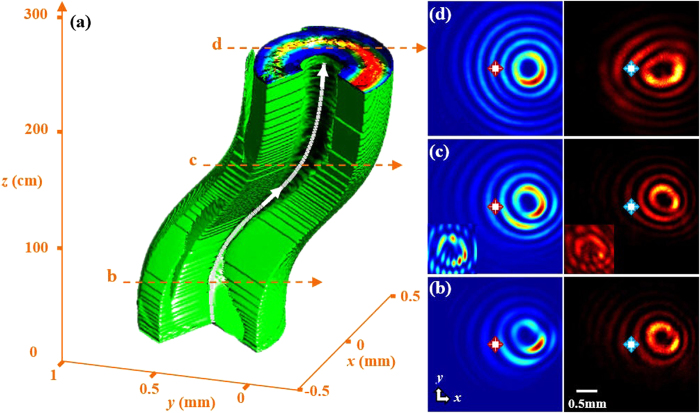
Demonstrations of a triply-charged singular beam (m = 3) propagating along a 3D curved trajectory. (**a**) Numerically simulated 3D visualization of the beam propagation, where the solid white curve represents the predesigned beam trajectory; (**b**–**d**) Numerically simulated (left column) and experimentally recorded (right column) beam patterns taken at different transverse planes marked in (**a**). The inserts in (**c**) are the corresponding interferograms, and the crosses mark the center of the initial beam as a reference point.

**Figure 4:**
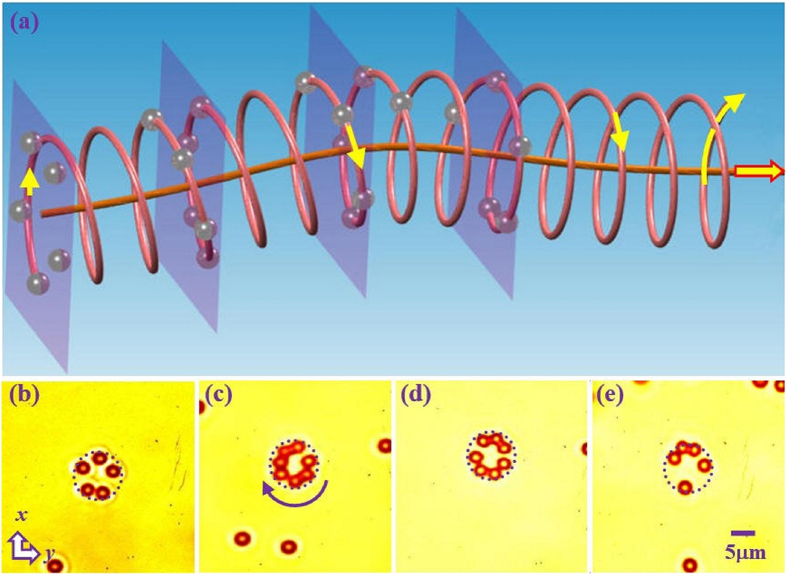
Experimental observation of 3D microparticle spiraling driven by a triply-charged singular beam propagating along a hyperbolic secant trajectory. (**a**) Schematic illustration of guiding and rotating particles along the bending trajectory; (**b–f**) Snapshots of trapped microparticles from videos taken when at different transverse planes. In each plane, the particles are spinning due to transfer of angular momentum from the beam (see Media 2 for an example), but the particles actually undergo spiral motion should they not be pushed against the holding glass. Dashed circle marks the main lobe of the singular beam.
