# Supplementary material for: Curved singular beams for three-dimensional particle manipulation
Source: Sci Rep. 2015 Jul 13;5:12086. doi: 10.1038/srep12086 (PMC4499814; doi:10.1038/srep12086)
Supplement: Supplementary Information [file srep12086-s1.pdf]

# – Supplementary Information –

## Curved singular beams for three-dimensional particle manipulation

**Juanying Zhao[1-3]*, Ioannis Chremmos[4,5]*, Daohong Song[1],**
**Demetrios N. Christodoulides[2], Nikolaos K. Efremidis[4], and Zhigang Chen[1,3]**

[1]*The MOE Key Laboratory of Weak-Light Nonlinear Photonics, and TEDA*

*Applied Physics Institute and School of Physics, Nankai University, Tianjin 300457, China*

[2]*CREOL/College of Optics, University of Central Florida, Orlando, Florida 32816*

[3]*Department of Physics and Astronomy, San Francisco State University, San Francisco, CA 94132*

[4]*Department of Mathematics and Applied Mathematics, University of Crete, Heraklion 71409, Greece*

[5]*Max Planck Institute for the Science of Light, Erlangen D-91058, Germany*

* *These authors made equal contribution.*

*zhigang@sfsu.edu*, *nefrem@uoc.edu*

### *S1: Media files*

The media files contain two short movies, illustrating the propagation dynamics of a self-accelerating singular beam and a case of particle manipulation using a beam of this kind. The first movie (Media 1) is an animation of the propagation dynamics of an $m$=1 Bessel-like vortex beam. Although the center is shifting and the main lobe exhibits some intensity "rotation" in the azimuthal direction, the size of the ring remains constant as the beam propagates. The second movie (Media 2) shows the observed spinning of traped microparticles in a transvsere plane by a triply-charged self-acceleating vortex beam propagating along a hyperbolic-secant trajectory. Note that the particles would undergo three-dimensional spiral motion should they have not been pushed against the holding glass.

(2 media files attached)

*S2: Detailed theoretical anaylsis of accelerating Bessel-like singular beams*

In the following we describe in detail the analysis leading to the design of accelerating singular beams of the higher-order Bessel type with arbitrary trajectories.

***Formulation of the problem***

We begin with the Fresnel integral of diffraction that describes the paraxial propagation of an optical wave **Error! Reference source not found.**

$$u(x,y,z)=\frac{1}{2\pi iz}\int_{-\infty}^{+\infty}\int_{-\infty}^{+\infty}u_0(\xi,\eta)\exp\left[iQ(\xi,\eta)+i\frac{(x-\xi)^2+(y-\eta)^2}{2z}\right]d\xi d\eta \qquad (1)$$

where the transverse coordinates $x,y,\xi,\eta$ are normalized by an arbitrary length $\ell$ and the propagation distance $z$ by $k\ell^2$ (equivalent to the Rayleigh length), while $k=2\pi/\lambda$ is the wavenumber. The amplitude and phase of the wave at the input plane $z=0$ is defined as

$$u(\xi,\eta,0)=u_0(\xi,\eta)\mathrm{e}^{iQ(\xi,\eta)} \qquad (2)$$

The main task of the analysis is to determine the phase $Q(\xi,\eta)$ that is required to produce an accelerating vortex beam with a given transverse width and a topological charge *m*. As a measure of the transverse width of the beam, we use the diameter of the inner low-intensity disk which is defined in Fig. S1.

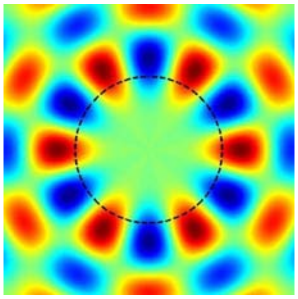

**Figure S1**: Real part of a vortex Bessel beam with order m = 6. Indicated is the inner low-intensity disk which is defined at the radius where the argument of the Bessel function is equal to its order.

We now employ ray optics. The equations of the rays follow from the condition of first-order stationarity of the function

$$P(\xi,\eta)=Q(\xi,\eta)+\frac{(x-\xi)^2+(y-\eta)^2}{2z} \qquad (3)$$

which is the total phase of the wave component contributed to the field point $(x,y,z)$ by the input point $(\xi,\eta,0)$. Setting the first-order partial derivatives of Eq.(3) equal to zero $(P_\xi = P_\eta = 0)$ we get

$$Q_\xi = \frac{x-\xi}{z}, \qquad Q_\eta = \frac{y-\eta}{z} \tag{4}$$

which are the equations of a ray from the input to the field point. Subsequently, we require that, at an arbitrary transverse plane $z$, the rays emitted at skew angles with respect to the $z$ axis from a (yet unknown) locus $L(z)$ on the input aperture pass from a circle with center $\mathbf{C}(z) = (f(z), g(z), z)$ and a fixed radius $r_m$. This circle we briefly denote as $(\mathbf{C}(z), r_m)$. The $m$ subscript indicates a connection of this radius with the order of vorticity. The functions $f(z), g(z)$ determine the trajectory of the beam with the propagation distance acting as a parameter. Using Eqs. (4), our requirement is expressed as

$$Q_\xi = \frac{f(z) + r_m \cos\varphi - \xi}{z}, \qquad Q_\eta = \frac{g(z) + r_m \sin\varphi - \eta}{z} \tag{5}$$

where $\varphi(\xi,\eta)$ is the azimuth angle of the point at which the ray from $(\xi,\eta,0)$ intersects the circle $(\mathbf{C}(z), r_m)$. The reference direction $(\varphi = 0)$ for measuring the azimuth is arbitrarily taken to be the $x$-axis. At this point we also note that, if any input point $(\xi,\eta,0)$ of its locus $L(z)$ is mapped to the distance $z$, then a two-variable function $z(\xi,\eta)$ is obtained. The latter is critical to finding $Q(\xi,\eta)$ but is yet unknown.

### ***Determination of the phase Q***

There are two key steps needed to proceed. The first is the requirement that the phase $Q(\xi,\eta)$ and its first two derivatives are continuous. A necessary requirement is that its mixed second-order partial derivatives should be equal, i.e. $Q_{\xi\eta} = Q_{\eta\xi}$, or using Eqs. (5)

$$(\xi - \xi_0 - r\cos\varphi) z_\eta - (zr\sin\varphi)\varphi_\eta = (\eta - \eta_0 - r\sin\varphi) z_\xi + (zr\cos\varphi)\varphi_\xi \tag{6}$$

where the subscripts $\xi,\eta$ imply the partial derivatives of the corresponding functions and

$$\xi_0(z) = f(z) - zf'(z), \qquad \eta_0(z) = g(z) - zg'(z). \tag{7}$$

Equation (6) is a differential one for the unknown functions $z(\xi,\eta)$ and $\varphi(\xi,\eta)$. We now assume that the locus $L(z)$ is a circle with center $(\xi_m(z),\eta_m(z),0)$ and radius $R(z)$, where all functions are to be determined explicitly

$$L(z):\quad (\xi-\xi_m(z))^2+(\eta-\eta_m(z))^2=R^2(z) \tag{8}$$

or equivalenty

$$\xi=\xi_m(z)+R(z)\cos\theta,\qquad \eta=\eta_m(z)+R(z)\sin\theta \tag{9}$$

where $\theta$ is the azimuth coordinate of the point $(\xi,\eta)$ on the circle $L(z)$. By differentiating Eq. (8) with respect to $\xi$ and $\eta$ we obtain the gradient $\nabla_{\xi,\eta}z$ as

$$\begin{aligned}\nabla_{\xi,\eta}z&=\left|\nabla_{\xi,\eta}z\right|(\cos\theta,\sin\theta)\,|\\ &=\frac{(\cos\theta,\sin\theta)}{\xi_m'(z)\cos\theta+\eta_m'(z)\sin\theta+R'(z)}\equiv\frac{(\cos\theta,\sin\theta)}{D(z,\theta)}\end{aligned} \tag{10}$$

where the prime denotes the derivative $d/dz$. From the obvious relation $\theta=\arctan\left(\frac{\eta-\eta_m(z)}{\xi-\xi_m(z)}\right)$ we also obtain the gradient $\nabla_{\xi,\eta}\theta$ as

$$\nabla_{\xi,\eta}\theta=\frac{\xi_m'(z)\sin\theta-\eta_m'(z)\cos\theta}{R(z)}\nabla_{\xi,\eta}z+\frac{(-\sin\theta,\cos\theta)}{R(z)} \tag{11}$$

The gradient $\nabla_{\xi,\eta}\varphi$ can also be obtained if we require that angles $\theta$ and $\varphi$ satisfy

$$\varphi=\theta+w(z) \tag{12}$$

where $w(z)$ is another function that is unknown for the moment. From the above equation we have

$$\nabla_{\xi,\eta}\varphi=\nabla_{\xi,\eta}\theta+w'(z)\nabla_{\xi,\eta}z\,. \tag{13}$$

Subsequently, we substitute Eqs. (9) into Eq. (6) and use Eqs. (10) to obtain:

$$\left[\frac{r_m}{R}\left(\xi_m'\cos w+\eta_m'\sin w\right)-u_m\right]\sin\theta+\left[\frac{r_m}{R}\left(\xi_m'\sin w-\eta_m'\cos w\right)+v_m\right]\cos\theta$$
$$+r_m\sin w\left(\frac{R'}{R}+w'\cot w-\frac{1}{z}\right)=0 \tag{14}$$

where

$$u_m(z)=\frac{\xi_m(z)-\xi_0(z)}{z},\qquad v_m(z)=\frac{\eta_m(z)-\eta_0(z)}{z} \tag{15}$$

Now notice that Eq. (14) is a trigonometric series for $\theta$ which holds for all $\theta\in[0,2\pi)$ if and only if all coefficients are zero. Hence

$$\begin{aligned}
&\xi_m'\cos w+\eta_m'\sin w=\frac{R}{r_m}u_m && (a)\\
&\xi_m'\sin w-\eta_m'\cos w=-\frac{R}{r_m}v_m && (b)\\
&\frac{R'}{R}+w'\cot w=\frac{1}{z} && (c)
\end{aligned} \tag{16}$$

Equation (16) is easily integrated to find

$$\sin\left(w(z)\right)=\frac{\beta z}{R(z)} \tag{17}$$

where $\beta>0$ is a real integration constant. By solving Eqs. (16) for $\xi_m'$ and $\eta_m'$ we obtain the first-order ODE system:

$$\begin{pmatrix}\xi_m'\\ \eta_m'\end{pmatrix}=\frac{R}{r_m}\begin{pmatrix}\cos w & -\sin w\\ \sin w & \cos w\end{pmatrix}\begin{pmatrix}u_m\\ v_m\end{pmatrix} \tag{18}$$

Alternatively, using Eqs. (15) and (17), the system becomes

$$\begin{pmatrix}u_m'\\ v_m'\end{pmatrix}=\frac{1}{r_m}\begin{pmatrix}\dfrac{R\cos w-r_m}{z} & -\beta\\ \beta & \dfrac{R\cos w-r_m}{z}\end{pmatrix}\begin{pmatrix}u_m\\ v_m\end{pmatrix}+\begin{pmatrix}f''\\ g''\end{pmatrix} \tag{19}$$

To solve this system, the radius $R(z)$ is required. This is found from the field profile in the neighbourhood of the center $\mathbf{C}(z)$. Within ray optics, the field profile can be determined approximately by assuming that each ray emitted from the circle $L(z)$ contributes a plane

wave $du$ in that region. Under the paraxial approximation and neglecting the variations of their amplitude, these elementary plane waves can be expressed as

$$du(z,\theta) \cong \exp\left( \begin{array}{l} iP(\xi,\eta) + i\dfrac{f + r_m \cos\varphi - \xi}{z}(\delta x - r_m \cos\varphi) \\ \qquad + i\dfrac{g + r_m \sin\varphi - \eta}{z}(\delta y - r_m \sin\varphi) \end{array} \right) d\theta \tag{20}$$

where $(\delta x, \delta y, 0) = (x, y, z) - (f, g, z)$ is the transverse displacement of the point of observation $(x, y, z)$ from $\mathbf{C}(z)$.

Also, $P(\xi,\eta)$ is the value of the total phase from the point $(\xi,\eta)$ on the circle $L(z)$ to the corresponding field point $(r_m,\varphi)$ on the circle $(\mathbf{C}(z), r_m)$. To find $P(\xi,\eta)$, we differentiate Eq. $P(\xi,\eta) = Q(\xi,\eta) + \dfrac{(x-\xi)^2 + (y-\eta)^2}{2z}$ (3) with respect to $\xi$ and $\eta$ and use the system (18) to find after some long algebraic calculations

$$P(\xi,\eta) = \frac{f - \xi_m}{z} r_m \cos\varphi + \frac{g - \eta_m}{z} r_m \sin\varphi + \beta r_m \varphi + W(z) \tag{21}$$

where

$$W(z) = \frac{1}{2}\int_0^z \left( f'^2 + g'^2 - u_m^2 - v_m^2 - \frac{r_m^2 + R^2 - 2Rr_m \cos w}{\zeta^2} \right) d\zeta \tag{22}$$

Now by inserting Eq. (9), (12) and (21) into Eq. (20) we obtain

$$\begin{aligned} du(z,\theta) &\cong \exp\left( iW + i\beta r_m w + ir_m \frac{r_m - R\cos w}{z} + i\frac{f - \xi_m}{z}\delta x + i\frac{g - \eta_m}{z}\delta y \right) \\ &\times \exp\left( i\beta r_m \theta + i\rho \frac{\sqrt{r_m^2 + R^2 - 2Rr_m \cos w}}{z} \cos(\theta - \mu + v(z)) \right) d\theta \end{aligned} \tag{23}$$

where $\delta x = \rho\cos\mu$ , $\delta y = \rho\sin\mu$ and $\nu(z) = \arcsin\left(\frac{r_m \sin w}{r_m \cos w - R}\right)$. Obviously $(\rho,\mu)$ are polar coordinates around the center $\mathbf{C}(z)$. Integrating all contributions over $\theta$, i.e. $\int_0^{2\pi} du$, we finally get

$$u(\rho,\theta,z) \cong 2\pi \exp\left(iW + i\beta r_m\left(w + \frac{\pi}{2} - \nu\right) + i r_m \frac{r_m - R\cos w}{z}\right)$$
$$\times \exp\left(i\frac{f-\xi_m}{z}\delta x + i\frac{g-\eta_m}{z}\delta y\right) J_{\beta r_m}\left(\rho\frac{\sqrt{r_m^2 + R^2 - 2Rr_m\cos w}}{z}\right)\exp(i\beta r_m \mu) \tag{24}$$

which reveals that the beam behaves locally around $\mathbf{C}(z)$ like a Bessel vortex of order $\beta r_m$, should we set

$$\beta r_m = m \tag{25}$$

Notice that, in order to have vortex beam that resists diffraction, the factor multiplying the polar radius $\rho$ in the argument of the Bessel function should be independent of $z$, namely $u \propto J_m(M\rho)$, or

$$\sqrt{r_m^2 + R^2 - 2Rr_m\cos w} = Mz \tag{26}$$

where the real constant $M > 0$ represents the (normalized) transverse wave number of the Bessel-like beam. Combining the above with Eq. (17), we finally obtain the radius

$$R(z) = \sqrt{M^2 z^2 + r_m^2 + 2r_m z\sqrt{M^2 - \beta^2}} \tag{27}$$

(Note that there is also a second solution $R(-z)$, which is however rejected because it is not monotonic with $z$ – the circles must be expanding).

At this point it would be interesting to comment that the result of Eq. (24) justifies our assumption of Eq. (8), namely, the rays are emitted from *circles* on the input plane. This is a general property of Bessel-type paraxial waves: such waves result from conical superpositions of rays emitted from circles on the input apertures.

Now that the radius $R(z)$ is known, functions $w(z)$, $P(\xi,\eta)$, $W(z)$ are readily determined from Eqs. (17), (21) and (22). The remaining functions $\xi_m(z),\eta_m(z)$ or alternatively $u_m(z),v_m(z)$ are determined by solving the system of Eq. (19). But before doing that, we need to relate the constants $M$ and $\beta$. To this end, we note that the rays emitted from a circle $L(z)$ create an oblique conical-like surface with a nonzero minimum waist (see Fig. S2). The minimum waist must be equal to $2r_m$. To see this more clearly, we express parametrically a ray starting from some point on the circle $L(z)$ with azimuth $\theta$

$$\mathbf{r}(\tau)=(1-\tau)\left(\xi_m+R\cos\theta,\eta_m+R\sin\theta,0\right)+\tau\left(f+r_m\cos\varphi,g+r_m\sin\varphi,z\right) \tag{28}$$

where $\tau$ is a dimensionless parameter, with $\mathbf{r}(0)$ being the starting point and $\mathbf{r}(1)$ the point on the circle $\left(\mathbf{C}(z),r_m\right)$ from which the ray passes, according to Eq. (5). It is easy to see from Eq. (28) that for any τ (hence at any propagation distance) the rays pass from a circle with center

$$\mathbf{C}(\tau)=(1-\tau)\left(\xi_m,\eta_m,0\right)+\tau\left(f,g,z\right) \tag{29}$$

and radius

$$\begin{aligned} r(\tau)&=\sqrt{\left((1-\tau)R\cos\theta+\tau r_m\cos\varphi\right)^2+\left((1-\tau)R\sin\theta+\tau r_m\sin\varphi\right)^2} \\ &=\sqrt{\left(R^2+r_m^2-2Rr_m\cos w\right)\tau^2-2R\left(R-r_m\cos w\right)\tau+R^2} \end{aligned} \tag{30}$$

Using Eqs. (17) and (26) we easily get from the above that $r_{\min}=\beta r_m/M$, which implies that we must have

$$M=\beta \tag{31}$$

We now proceed to solve the system (19), which, due to Eq. (31), simplifies to

$$\begin{pmatrix} u_m' \\ v_m' \end{pmatrix}=\frac{1}{r_m}\begin{pmatrix} 0 & -\beta \\ \beta & 0 \end{pmatrix}\begin{pmatrix} u_m \\ v_m \end{pmatrix}+\begin{pmatrix} f'' \\ g'' \end{pmatrix} \tag{32}$$

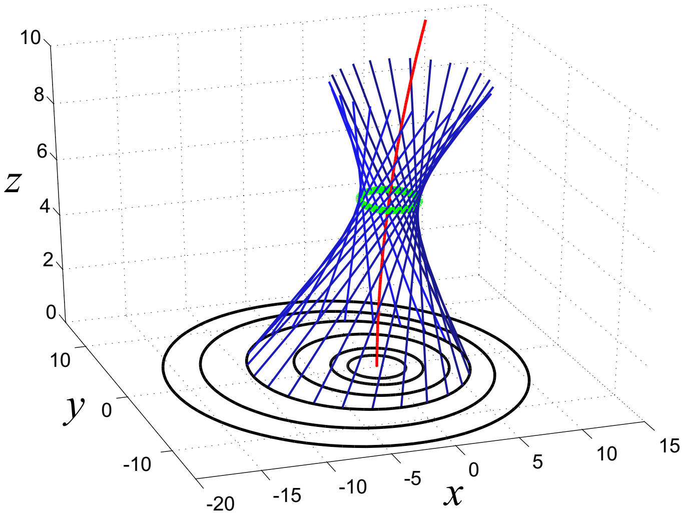


**Figure S2**: Schematic of the principle. Rays emitted from expanding circles on the input plane and at skew angles with respect to the *z* axis interfere to create an oblique cone-like surface with a minimum waist diameter equal to $2r_m$ (green circle). The field around the minimum waist is proportional to a vortex Bessel wavefunction of order *m*.

However, the initial condition that is required to solve this system cannot be determined from Eq. (15) by simply letting $\xi_m(0)=\eta_m(0)=0$, because we also have $\xi_0(0)=\eta_0(0)=0$, thus getting an indefinite ratio 0/0. For this reason we think as follows: the circles $L(z)$ must be expanding and never intersecting. This means that the denominator $D(z,\theta)$ in Eq. (10) must be nonnegative for all $\theta$ which happens if and only if

$$R'(z)\geq\sqrt{\xi_m'^2(z)+\eta_m'^2(z)}=\frac{R(z)}{r_m}\sqrt{u_m^2(z)+v_m^2(z)} \tag{33}$$

for all $z$, where we have used Eq. (18) to replace the functions $\xi_m,\eta_m$ by $u_m,v_m$. Setting in the above $z=0$ and since $R'(0)=0$, $R(0)=r_m$ from Eq. (27) and (31), we get $\sqrt{u_m^2(0)+v_m^2(0)}\leq 0$ namely $u_m(0)=v_m(0)=0$. Now the functions $u_m,v_m$ can be completely determined from the system (32) once the trajectory functions $f,g$ are given. Subsequently, the functions $\xi_m,\eta_m$ are obtained from Eqs. (15).

***Algorithm for computing the phase Q***

In the following, we outline the main procedure for computing the phase function $Q(\xi,\eta)$. Given are the order $m$ of the singular beam, its minimum radius $r_m$ (or alternatively its transverse wavenumber $\beta = m/r_m$) and the trajectory functions $f(z), g(z)$. There are four steps:

1) For any point $(\xi,\eta,0)$ on the input plane, solve Eq. (8) for $z$ to find the unique circle $L(z)$ passing from this point.

2) Compute $W(z)$ from Eq. (22), which now simplifies to

$$W(z) = \frac{1}{2}\int_0^z \left(f'^2 + g'^2 - u_m^2 - v_m^2\right) d\zeta - \frac{1}{2}\beta^2 z \tag{34}$$

where functions $u_m(z), v_m(z)$ have been determined from solving the system of Eq. (32).

3) Compute $P(\xi,\eta)$ from Eq. (21), where the phase $\varphi$ is computed from Eq. (12) and $w(z)$ from Eq. (17), with $\theta = \arctan\left(\frac{\eta - \eta_m(z)}{\xi - \xi_m(z)}\right)$.

4) Finally, obtain $Q(\xi,\eta)$ from Eq. $P(\xi,\eta) = Q(\xi,\eta) + \frac{(x-\xi)^2 + (y-\eta)^2}{2z}$ (3) as

$$Q(\xi,\eta) = P(\xi,\eta) - \frac{(f + r_m\cos\varphi - \xi)^2 + (g + r_m\sin\varphi - \eta)^2}{2z} \tag{35}$$

***Example: A vortex beam with constant acceleration***

As an example, let us consider the case of a singular beam with the 2D (lying on the *xz* plane) parabolic trajectory: $f(z) = \gamma z^2$, $g(z) = 0$. The system (32) is exactly solvable and the solution reads in terms of the center coordinates $\xi_m, \eta_m$ as

$$\xi_m(z) = -\gamma z^2 + \frac{2\gamma r_m}{\beta} z \sin\left(\frac{\beta z}{r_m}\right), \qquad \eta_m(z) = \frac{2\gamma r_m}{\beta} z \left[1 - \cos\left(\frac{\beta z}{r_m}\right)\right] \tag{36}$$

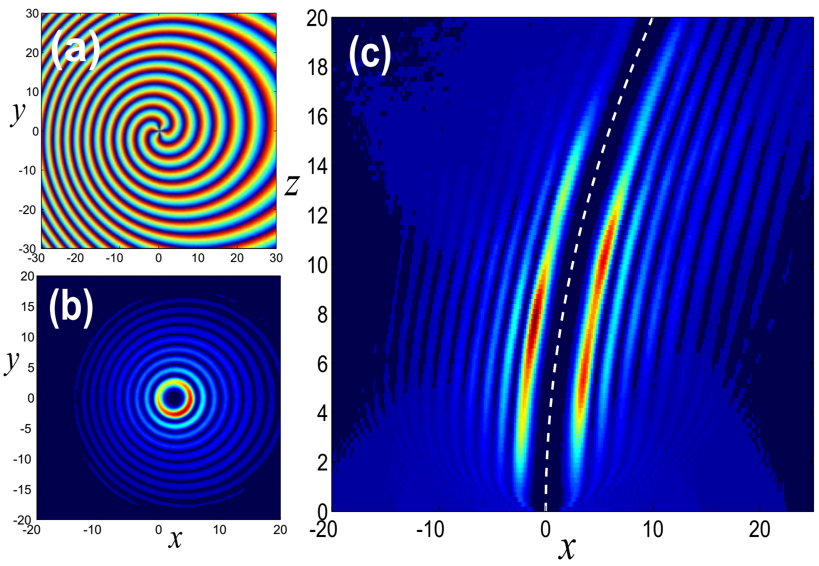


**Figure S3**: (a) Input phase, (b) beam profile at $z=10$ and (c) intensity cut on $y=0$ for the vortex beam with order $m=4$, diameter $2r_m=4$ and parabolic trajectory $x=z^2/40$ (dotted curve).

Inserting the above into Eq. (34), we also find exactly

$$W(z)=\frac{2}{3}\gamma^2 z^3-\left(\frac{4\gamma^2 r_m^2}{\beta^2}+\frac{\beta^2}{2}\right)z+\frac{4\gamma^2 r_m^3}{\beta^3}\sin\left(\frac{\beta z}{r_m}\right) \tag{37}$$

Following the algorithm described in the previous paragraph and using the last two equations, we are able to compute the input phase, noting that the only numerical part of the procedure is the solution of Eq. (8) for $z$ through the Newton-Raphson method. An example is shown in Fig. S3 in terms of the input phase and intensity snapshots of the beam. The ray structure for this example is shown in Fig. S2.

***A final note***

The condition Eq. (33) is a prerequisite for computing the phase through the presented method, ensuring that the circles of Eq. (8) are expanding but never intersecting each other. However, for trajectories whose acceleration does not approach to zero as $z\to\infty$, this condition is satisfied only for distances below a certain bound, or $z\le z_{\max}$. Beyond this distance, a new trajectory must be defined in order to satisfy the condition, as for example a straight line. The procedure is then similar to that of the zero-order Bessel beams discussed previously in Ref 2.

***References***

[1] Goodman J., Introduction To Fourier Optics (Roberts and Company Publishers, 2005)

[2] Chremmos I. D., Chen Z., Christodoulides D. N. & Efremidis N. K. Bessel-like optical beams with arbitrary trajectories. Optics Letters 37, 5003-5005, 2012.
